# An Exploratory Comparison of Pilates and Weight Circuit Training on Body Composition, Pelvic Alignment, and Balance in Obese Middle-Aged Women

**DOI:** 10.3390/jfmk11020141

**Published:** 2026-03-27

**Authors:** Du-Hwan Oh, Jang-Kyu Lee

**Affiliations:** 1 Korea Exercise Medicine & Science Association, 93-3, Bansong-dong, Dongtan-gu, Hwaseong-si 18454, Gyeonggi, Republic of Korea; alzl10000@naver.com; 2 Department of Exercise Prescription & Rehabilitation, Dankook University, 119, Dandae-ro, Dongnam-gu, Cheonan-si 31116, Chungnam, Republic of Korea

**Keywords:** obesity, pelvic alignment, Pilates circuit training, weight circuit training, dynamic balance, static balance

## Abstract

**Background:** Middle-aged women with obesity frequently exhibit postural misalignment and impaired balance control, which may increase the risk of functional limitations and falls. This study aimed to compare the effects of Pilates circuit training and weight circuit training on body composition, pelvic alignment indices, and balance performance in obese middle-aged women. **Methods:** Eighteen women (body fat ≥ 30%) were randomized to either a Pilates circuit training group (PCG, *n* = 9) or a weight circuit training group (WCG, *n* = 9) in an exploratory comparative study. Both groups performed supervised exercise three times per week for eight weeks. Outcome measures included body composition, pelvic alignment indices, dynamic balance (Y-Balance Test), and static balance (BESS). Data were analyzed using a two-way mixed ANOVA to examine time, group, and interaction effects. **Results:** Both groups showed significant reductions in body weight (PCG: −3.09 kg; WCG: −2.00 kg), percentage body fat (PCG: −1.85%; WCG: −1.53%), and waist-to-hip ratio (PCG: −0.05; WCG: −0.04) (*p* < 0.01). Significant improvements in pelvic alignment indices were observed primarily in the PCG, whereas the WCG showed smaller changes. Dynamic and static balance improved in both groups, with greater improvements observed in the PCG. **Conclusions:** Both training modalities improved body composition and balance outcomes in obese middle-aged women. Pilates circuit training may be associated with greater improvements in pelvic alignment and balance; however, these findings should be interpreted cautiously due to the exploratory design and small sample size. Further adequately powered randomized controlled trials are required to confirm these findings.

## 1. Introduction

Middle-aged women with obesity experience various physiological changes, including increased body fat accumulation, decreased muscular strength and endurance, impaired balance ability, and reduced flexibility [1]. Obesity in this population has been associated with alterations in pelvic tilt angles and impaired postural control, increasing the risk of injuries and falls and predisposing individuals to chronic musculoskeletal disorders [2].

Physiological and metabolic changes associated with aging may lead to gradual alterations in body composition in middle-aged women, including changes in fat distribution. Excess body weight is a well-established risk factor for metabolic syndrome and chronic diseases such as cardiovascular disease, type 2 diabetes, and hypertension [3]. Exercise has been widely recommended as an effective intervention for improving body composition and mitigating obesity-related complications. Both Pilates- and resistance-based training have been used to improve body composition, muscular fitness, and postural function in adult populations [4,5]. However, their comparative effects on pelvic alignment and balance in middle-aged women with obesity remain insufficiently understood.

Obesity-induced postural imbalances, including pelvic asymmetry, rotation, and tilt, have been identified as contributing factors to musculoskeletal dysfunction [6]. Pelvic misalignment in individuals with obesity may present in various forms, such as anterior–posterior tilt, transverse rotation, and lateral inclination. These alterations are often associated with imbalances in trunk and hip musculature, including the abdominal muscles, erector spinae, gluteal muscles, and hip stabilizers. Such muscular imbalances may alter lumbopelvic mechanics and increase loading on the lumbar spine. Over time, these changes may influence spinal alignment, postural control, and movement efficiency, and may also be associated with an increased risk of musculoskeletal discomfort and functional limitations. Among these, pelvic tilt is of particular concern, as disproportionate fat distribution and increased lumbar loading may be linked to postural misalignment and low back pain [7]. In addition, excessive body weight may affect pelvic rotation and inclination, further contributing to postural instability and impaired balance control [8].

Both dynamic and static balance are closely related to pelvic alignment. Middle-aged women with obesity have been reported to exhibit impaired balance ability, which may increase susceptibility to falls. Previous studies suggest that improvements in pelvic alignment may be associated with enhanced balance control, highlighting the importance of postural alignment in functional stability [9].

However, comparative evidence regarding the effects of Pilates and resistance-based circuit training on pelvic alignment and balance in middle-aged women with obesity remains limited. Therefore, the present study aimed to compare the effects of Pilates circuit training and weight circuit training on body composition, pelvic alignment indices, and balance performance in middle-aged women with obesity. This study was conducted as an exploratory comparative investigation to provide preliminary effect size estimates and to inform the design of future confirmatory studies.

## 2. Materials and Methods

### 2.1. Participants

In this exploratory comparative study, a total of 18 women aged ≥ 45 years with a body fat percentage of ≥30% were recruited and randomized to the Pilates circuit training group (PCG, *n* = 9) or the weight circuit training group (WCG, *n* = 9). All participants were classified as physically inactive, defined as not engaging in regular structured exercise for at least six months prior to the study. Participants were excluded if they had a history of musculoskeletal injuries or surgery within the past six months, cardiovascular or metabolic diseases that contraindicated exercise, or neurological disorders affecting balance. After eligibility screening, participants were randomized (1:1) using a computer-generated sequence, and allocation was concealed in sequentially numbered, opaque, sealed envelopes. Given the exploratory nature of this study, no formal a priori sample size calculation was performed. Instead, the sample size was determined pragmatically based on recruitment feasibility and the capacity to supervise the interventions. The primary purpose of this study was not to provide confirmatory evidence, but to generate preliminary effect size estimates that could inform the design and sample size planning of future confirmatory trials. Participant blinding was not feasible due to the nature of exercise interventions. However, efforts were made to minimize potential bias by standardizing testing procedures and providing consistent instructions to all participants. Outcome assessments were conducted by the same research staff involved in the intervention delivery, and assessor blinding was not implemented. This should be considered when interpreting the study findings. This study was approved by the Institutional Review Board of Catholic Kwandong University (IRB No. CKU-24-02-1808) and was conducted in accordance with the Declaration of Helsinki. All participants provided written informed consent prior to participation after receiving a detailed explanation of the study procedures. The physical characteristics of the participants are presented in Table 1.

### 2.2. Training Program

Pilates and weight circuit training were performed three times per week for 8 weeks, with each session lasting approximately 45 min (warm-up 5–10 min, main exercise 30 min, cool-down 5 min). The main exercise phase was structured as a circuit training program consisting of multiple exercises targeting the upper body, lower body, and core muscle groups. Participants completed each exercise in sequence with minimal rest between stations to maintain continuous engagement.

To ensure progressive overload, the initial training intensity was set at approximately 20 repetitions maximum (20 RM), corresponding to a moderate-intensity resistance level. Training loads were re-evaluated and adjusted every two weeks to reflect improvements in individual strength and to maintain the target relative intensity throughout the intervention period [10,11] (Table 2).

Exercise intensity was standardized between the Pilates circuit training and weight circuit training groups using both objective and subjective monitoring methods. Heart rate (HR) was continuously monitored using the Polar Pacer Pro (Polar Electro Oy, Kempele, Finland), and target heart rate was calculated using the Karvonen formula: (HRmax − HRrest) × %intensity + HRrest. Maximum heart rate (HRmax) was estimated using an age-based predictive equation (220 − age), and resting heart rate (HRrest) was measured after 10 min of seated rest prior to testing. Heart rate reserve (HRR) was calculated as HRmax − HRrest. Exercise intensity was maintained within a moderate-intensity range (60–75% HRR), corresponding to a moderate-intensity range.

In addition, rating of perceived exertion (RPE) was assessed using the Borg scale (6–20). Participants reported their RPE at the end of each session, and exercise intensity was adjusted when RPE values fell outside the target range of 12–15. The combined use of HR and RPE allowed for individualized regulation of exercise intensity and improved control of internal training load.

Adherence to the intervention was monitored throughout the 8-week program by recording attendance at each supervised training session. Compliance was calculated as the percentage of completed sessions relative to the total prescribed sessions. Mean attendance exceeded 95% in both groups, indicating high adherence to the intervention.

### 2.3. Body Composition

Body composition was assessed using bioelectrical impedance analysis (BIA) with an InBody 280 device (InBody Co., Ltd., Seoul, Republic of Korea). Prior to measurement, participants were instructed to refrain from food intake for at least 2 h and avoid alcohol consumption for a minimum of 24 h to ensure accurate readings. Menstrual cycle phase was not controlled or recorded during pre- and post-intervention assessments; therefore, potential fluid-related variability associated with hormonal fluctuations cannot be excluded. During the measurement, participants were required to remove their shoes and socks, place their feet correctly on the electrodes, and maintain an upright posture. They were also instructed to grip the hand electrodes firmly with both hands while keeping their arms extended away from the torso until the measurement was completed.

### 2.4. Pelvic Alignment Analysis

Pelvic alignment was assessed using a three-dimensional (3D) body shape analysis system (Moti Physio; MG Solutions, Seoul, Republic of Korea), which utilizes optical scanning technology to capture spatial coordinates of anatomical landmarks. The system reconstructs a 3D model of the participant’s posture and calculates pelvic alignment variables based on predefined reference points, including the bilateral iliac crests and pelvic landmarks. All measurements were performed with participants standing in a standardized anatomical position. The output variables, including pelvic tilt, rotation, and inclination, were expressed in degrees (°) and were interpreted as functional pelvic alignment indices derived from three-dimensional positional differences between anatomical landmarks. These variables do not represent pure anatomical movements within a single biomechanical plane (e.g., sagittal, frontal, or transverse), and the term “pelvic tilt” in this study should therefore be interpreted as a functional postural index rather than a direct measure of sagittal pelvic tilt. Previous studies have demonstrated that 3D optical scanning systems provide reliable and non-invasive assessments of postural alignment in clinical and research settings [2].

The present study focused specifically on pelvic alignment variables derived from three-dimensional postural analysis. A comprehensive static postural assessment of the entire body (e.g., spinal curvature, head position, or lower limb alignment) was not performed. Therefore, it should be acknowledged that pelvic misalignment may be associated with broader postural deviations, and the absence of whole-body postural assessment limits the ability to fully interpret the relationship between pelvic alignment and overall posture. This should be considered when interpreting the study findings [12].

### 2.5. Pelvic Tilt

In the present study, the variable labeled pelvic tilt was operationally derived from three-dimensional positional differences between predefined anatomical landmarks and should be interpreted as a functional postural index rather than a direct anatomical measure of sagittal pelvic tilt. The Moti Physio system identifies bilateral pelvic landmarks and calculates the relative vertical displacement between anterior and posterior reference points to determine the direction and magnitude of pelvic tilt. The pelvic tilt angle (°) was computed based on the inclination of the pelvic segment relative to the vertical axis, with positive values indicating anterior tilt and negative values indicating posterior tilt. The absolute value of the angle was used for analysis to represent the magnitude of deviation from neutral alignment.

### 2.6. Pelvic Rotation

Pelvic rotation was defined as the rotational displacement of the pelvis in the transverse plane. The Moti Physio system calculates pelvic rotation by comparing the spatial orientation of anterior pelvic landmarks (e.g., anterior superior iliac spine) and posterior reference points. The rotation angle (°) was derived from the angular difference between the left and right pelvic landmarks relative to the body’s longitudinal axis. Higher absolute values indicate greater asymmetry in pelvic rotation, reflecting increased deviation from neutral alignment.

### 2.7. Pelvic Inclination

Pelvic inclination was defined as the lateral tilt of the pelvis in the frontal plane. This variable was calculated based on the angular difference between the left and right iliac crests relative to a horizontal reference line. The inclination angle (°) represents the degree of asymmetry between both sides of the pelvis, with larger absolute values indicating greater lateral imbalance. All measurements were obtained under standardized standing conditions to minimize postural variability.

### 2.8. Y-Balance Test (Dynamic Balance Test)

**Figure 1:**
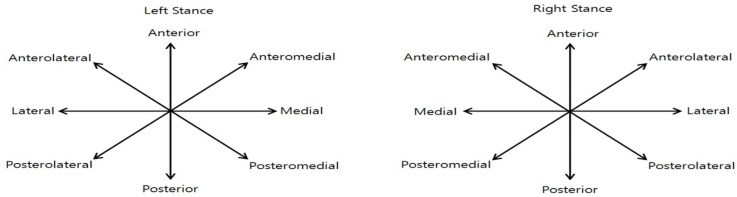
SEBT directions. Directions of the Star Excursion Balance Test (SEBT): anterior, posterolateral, and posteromedial.

**Figure 2:**
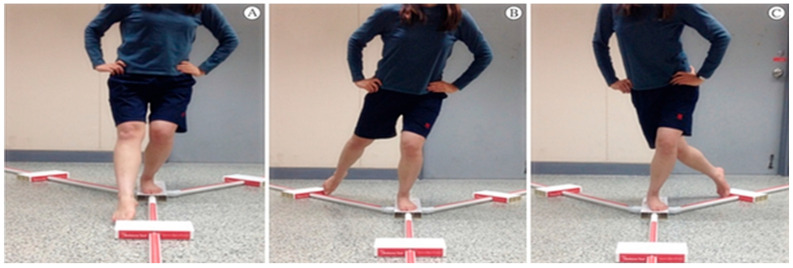
Y-Balance Position. Position adopted during the Y-Balance Test. (**A**), Anterior; (**B**), posterolateral; (**C**), posteromedial.

Dynamic balance ability was assessed using the Y-Balance Kit, measuring the reach distances in three directions: anterior, posteromedial, and posterolateral (Figure 1 and Figure 2). Participants positioned the center of their foot on the center of the grid and performed three practice trials before completing three test trials in each direction. The average value of the three trials was recorded. The mean reach distance for each direction was used in subsequent normalization and composite score calculations. To minimize fatigue, a 30 s rest period was provided between trials in different directions [13]. Since reach distance can vary depending on individual height, leg length was measured from the anterior superior iliac spine (ASIS) to the medial malleolus to normalize the results [14]. The following equation was used for normalization:

Normalized reach (%) = (excursion distance/leg length) × 100

Finally, dynamic balance ability was standardized using the following equation [13]:

Composite score (%) = (anterior + posteromedial + posterolateral excursion distance)/(3 × leg length) × 100

In this equation, anterior, posteromedial, and posterolateral represent the mean reach distances obtained from the three test trials in each direction.

### 2.9. Balance Error Scoring System (BESS) Test (Static Balance Test)

**Figure 3:**
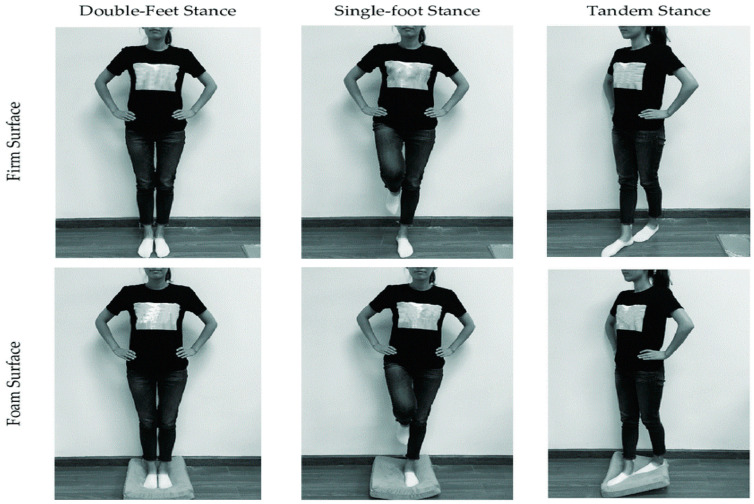
BESS test Position. Position adopted during the Balance Error Scoring System (BESS) test.

Static balance was assessed using the Balance Error Scoring System (BESS), a standardized and widely used clinical tool for evaluating postural stability [15]. The BESS protocol consists of six testing conditions, each performed for 20 s with the participant’s eyes closed and hands placed on the iliac crests. The six conditions include three stance positions (double-leg stance, single-leg stance on the non-dominant limb, and tandem stance with the non-dominant foot positioned posteriorly), performed on two different surfaces (firm and foam) (Figure 3). Participants were instructed to maintain each position as steadily as possible throughout the testing period.

### 2.10. Error Classification

A postural error was defined as any observable deviation from the standardized testing position during each 20 s trial. Errors were classified according to established BESS criteria and included the following:

(1) Opening the eyes
(2) Lifting the hands off the iliac crests
(3) Stepping, stumbling, or falling
(4) Moving the hip into more than 30° of flexion or abduction
(5) Lifting the forefoot or heel off the testing surface
(6) Remaining out of the testing position for more than 5 s

All errors were recorded by a trained evaluator during each trial, and only clearly observable deviations were counted to ensure scoring consistency.

### 2.11. Scoring

Each error was assigned a value of one point, and the total BESS score was calculated as the sum of all errors across the six conditions. A maximum of 10 errors was recorded per condition, resulting in a total possible score ranging from 0 to 60. Higher scores indicate poorer postural stability, whereas lower scores reflect better balance control. For analytical purposes, the total BESS score was treated as a continuous variable, with changes in score interpreted as improvements or declines in static balance performance.

All outcome assessments were conducted by a trained evaluator following standardized measurement protocols to ensure consistency across testing sessions. Prior to data collection, the evaluator was familiarized with all assessment procedures. However, formal intra- and inter-rater reliability were not quantified in the present study, and this should be considered when interpreting the findings, as measurement variability cannot be entirely excluded.

To mitigate potential assessor bias, standardized scoring criteria were strictly applied, and all assessments were conducted following a consistent protocol across all participants and time points. However, because assessor blinding was not feasible, the possibility of detection bias cannot be fully excluded.

The order of all performance tests was fixed and standardized across all participants to ensure consistency across measurements.

### 2.12. Statistics

All statistical analyses were performed using SPSS Statistics version 25 (IBM Corp., Armonk, NY, USA). Continuous variables are presented as means and standard deviations. A two-way mixed analysis of variance (ANOVA) with repeated measures on time (Week 0 vs. Week 8) was conducted to examine the main effects of group (PCG vs. WCG), time, and their interaction. When a significant interaction was detected, simple effects analyses were performed, and multiple comparisons were adjusted using the Bonferroni correction. Within-group pre–post changes were additionally examined using paired *t*-tests as supplementary analyses. Assumptions of normality (Shapiro–Wilk test) and homogeneity of variance (Levene’s test) were verified. All tests were two-tailed, with the level of statistical significance set at α = 0.05.

Effect sizes for within-group changes were calculated using Cohen’s d (Morris’s d_av for pre–post designs) [16]. For ANOVA effects, partial eta-squared (partial η^2^) was reported [17]. The magnitude of Cohen’s d was interpreted as small (0.2), moderate (0.5), and large (≥0.8) according to conventional thresholds [17]. Effect sizes are reported with their original sign to reflect the direction of change, and both the magnitude and direction (increase or decrease) are described in the text. Given the exploratory nature of the present study and the relatively small sample size, greater emphasis was placed on descriptive statistics and the magnitude of effects, including the interpretation of effect sizes alongside their variability, rather than on sole reliance on null hypothesis significance testing.

## 3. Results

To provide additional information on the magnitude of change, absolute pre–post differences (Δ) were calculated for each outcome variable and were considered when interpreting within-group changes alongside the inferential statistical results.

### 3.1. Body Composition

Both groups showed significant improvements over time in several body composition variables. Body weight, fat mass, percentage body fat, and waist-to-hip ratio decreased significantly following the 8-week intervention, as reflected by the mean changes and corresponding 95% confidence intervals presented in Table 3, whereas lean body mass showed no consistent pattern of change between groups despite a statistically significant within-group change observed in the Pilates circuit training group. Although the magnitude of change tended to be greater in the Pilates circuit training group for selected adiposity-related variables, the between-group differences in mean change and their corresponding 95% confidence intervals indicated that no significant group × time interaction effects were observed for body composition outcomes. Detailed descriptive values, including mean changes (Δ) and corresponding 95% confidence intervals, are presented in Table 3, while between-group effect sizes and supplementary analyses are provided in Supplementary Table S1.

### 3.2. Pelvic Alignment

Pelvic alignment variables (pelvic tilt, rotation, and inclination) were calculated as angular deviations (°) derived from three-dimensional positional differences between predefined anatomical landmarks, as described in Section 2.4. Absolute values were used for analysis to represent the magnitude of asymmetry, with higher values indicating greater deviation from neutral pelvic alignment.

Significant group × time interaction effects were observed for pelvic tilt, pelvic rotation, and pelvic inclination (Table 4). In the Pilates circuit training group, pelvic tilt (Δ = −1.53°, 95% CI: −1.98 to −1.08), pelvic rotation (Δ = −1.47°, 95% CI: −2.06 to −0.87), and pelvic inclination (Δ = −2.24°, 95% CI: −3.36 to −1.13) showed significant reductions, whereas changes in the weight circuit training group were smaller and not statistically significant for several variables. Improvements in pelvic alignment indices were consistently greater in the Pilates circuit training group, whereas the weight circuit training group showed smaller changes, with limited or non-significant improvement in some variables. These findings indicate that the two interventions elicited different patterns of change in pelvic alignment. The between-group differences at post-intervention were −0.93° (95% CI: −1.52 to −0.35, *p* = 0.004) for pelvic tilt, −0.77° (95% CI: −1.35 to −0.18, *p* = 0.013) for pelvic rotation, and −1.29° (95% CI: −2.39 to −0.19, *p* = 0.024) for pelvic inclination. Detailed descriptive values are presented in Table 4, and additional statistical results, including partial η^2^ values, are provided in Supplementary Table S2. The direction and magnitude of change were interpreted based on the mean differences (Δ) and their corresponding 95% confidence intervals.

### 3.3. Balance

Significant improvements in both dynamic and static balance were observed following the intervention (Table 5). For the Y-Balance Test, both groups demonstrated improved performance over time, with greater gains observed in the Pilates circuit training group and significant interaction effects in selected directions. In the Pilates circuit training group, Y-balance performance increased by 11.53% (95% CI: 10.51 to 12.56) on the right side and 11.13% (95% CI: 10.05 to 12.21) on the left side, whereas the weight circuit training group showed smaller increases of 9.19% (95% CI: 8.48 to 9.91) and 8.02% (95% CI: 6.88 to 9.16), respectively. However, the within-group effect size estimates for the Y-Balance outcomes were notably large and should be interpreted with caution. These values are likely influenced by the relatively small variability in the normalized reach scores and the use of standardized scaling, which may inflate effect size estimates in small samples, particularly when variability is low. The relatively small standard deviations observed in the Y-Balance Test likely reflect low within-group variability associated with normalized reach scores rather than measurement error. Therefore, greater emphasis should be placed on the direction and statistical significance of change rather than on the absolute magnitude of effect sizes. In line with the exploratory design, effect size estimates should be interpreted together with their associated variability, and descriptive patterns of change are emphasized over strict dichotomous interpretation based on *p*-values. For the BESS, error scores decreased in both groups, indicating improved static balance, and descriptively greater improvement was observed in the Pilates circuit training group. In the Pilates circuit training group, BESS error scores decreased by −2.67 (95% CI: −3.44 to −1.90) on the firm surface and −4.44 (95% CI: −5.22 to −3.67) on the foam surface, whereas the weight circuit training group showed smaller reductions of −1.44 (95% CI: −1.85 to −1.04) and −3.33 (95% CI: −4.77 to −1.90), respectively. The between-group differences at post-intervention were 2.23 (95% CI: 0.70 to 3.75, *p* = 0.007) for Y-balance (right), 2.95 (95% CI: 1.44 to 4.47, *p* = 0.001) for Y-balance (left), −0.56 (95% CI: −1.50 to 0.39, *p* = 0.229) for BESS firm, and −1.22 (95% CI: −2.45 to 0.01, *p* = 0.051) for BESS foam. Detailed descriptive values and supplementary statistical results are presented in Table 5 and Supplementary Table S2.

## 4. Discussion

### 4.1. Body Composition

Exercise interventions are well recognized as effective strategies for improving body composition by reducing body fat and preserving muscle mass, thereby contributing to obesity prevention and management [18]. In the present study, both the Pilates circuit training group and the weight circuit training group demonstrated significant improvements in body composition variables, including decreases in body weight, body fat mass, body fat percentage, and waist-to-hip ratio. However, the magnitude of change appeared greater in the PCG compared with the WCG, particularly for body fat mass and WHR. These findings may indicate a tendency toward comparatively greater improvements in central adiposity indicators following Pilates circuit training.

Pilates has been suggested to facilitate activation of deep core musculature and to influence neuromuscular coordination, which may be associated with improvements in exercise efficiency and energy expenditure; however, these mechanisms were not directly assessed in the present study [19]. Recent studies have also indicated that regular Pilates participation contributes to reductions in abdominal obesity and improvements in WHR through mechanisms related to enhanced trunk stability and fat oxidation [20]. The present findings are consistent with Cancela et al. [4], who observed decreases in fat mass and improvements in body composition following Pilates exercise in elderly women. Moreover, the results align with Wells et al. [21], who emphasized that the integrative nature of Pilates, combining strength, flexibility, and postural control, may provide a unique advantage in improving obesity-related indicators.

On the other hand, resistance-based circuit training such as WCG plays a pivotal role in increasing lean body mass (LBM), which enhances basal metabolic rate [22]. The current results indicated that although body fat reduction was more evident in the PCG, participants in the WCG maintained relatively higher levels of LBM. This is in line with previous research reporting that resistance training stimulates hypertrophy of large muscle groups, facilitates muscle mass preservation, and contributes to fat oxidation [5]. Taken together, these findings suggest that both exercise modalities may contribute to improvements in body composition in middle-aged women with obesity. Pilates circuit training may indicate a tendency toward greater improvements in central adiposity indicators, whereas weight circuit training may be associated with the preservation of lean body mass. However, these modality-specific differences should be interpreted cautiously given the exploratory design and limited sample size. The observed changes should also be interpreted in light of the reported mean differences (Δ) and their corresponding 95% confidence intervals, which provide an estimate of the magnitude and precision of the effects.

### 4.2. Pelvic Alignment

Several previous studies have highlighted the role of Pilates in improving postural and pelvic alignment by targeting deep trunk musculature such as the transverse abdominis and multifidus [21,23]. In the present study, significant improvements were observed in pelvic tilt, pelvic rotation, and pelvic inclination following 8 weeks of Pilates circuit training, with large to very large effect sizes (Cohen’s d = 2.11–2.57). However, these magnitude estimates should be interpreted with consideration of the exploratory design and limited sample size. These findings are consistent with prior reports suggesting that Pilates-based interventions may influence spinal and pelvic alignment and reduce musculoskeletal strain [19,21].

Obesity-related factors, including excessive body weight and altered fat distribution, have been associated with changes in lumbopelvic posture [2,7]. The present results suggest that Pilates circuit training may be associated with favorable changes in pelvic alignment indices, potentially through adaptations related to trunk muscle activation and flexibility; however, these mechanisms remain speculative and were not directly measured in this study. From a biomechanical perspective, the observed differences between Pilates circuit training and weight circuit training may be related to the distinct movement characteristics and neuromuscular demands of each modality. Pilates-based exercises typically emphasize controlled, multi-planar movements with a strong focus on trunk stabilization, segmental control, and coordinated activation of deep core musculature, which may contribute to improved postural alignment and dynamic balance. In contrast, weight-based circuit training generally involves higher external loads and predominantly linear movement patterns, which may preferentially enhance muscle strength and hypertrophy but may provide relatively less stimulus for fine postural control and intersegmental coordination. These modality-specific characteristics may partially explain the comparatively greater improvements observed in pelvic alignment and balance in the Pilates circuit training group; however, these interpretations remain hypothetical, as neuromuscular activation patterns and kinematic variables were not directly assessed in the present study. These interpretations are supported by previous studies demonstrating that core-focused and stabilization-based exercises enhance postural control and balance through improved neuromuscular coordination [24,25]. Caution is warranted when inferring physiological mechanisms from outcome measures alone, as such interpretations may extend beyond the directly observed data [26]. Supplementary analyses demonstrated consistent trends across multiple alignment measures, although these observations require confirmation in larger samples.

Although alterations in pelvic alignment have been linked to musculoskeletal discomfort and balance impairments [27], the current findings should be considered preliminary. Further controlled studies are necessary to determine whether the observed changes translate into clinically meaningful functional benefits.

Overall, these exploratory findings indicate that Pilates circuit training may be associated with changes in functional pelvic alignment indices in middle-aged women with obesity. However, definitive conclusions regarding clinical applicability or therapeutic effectiveness cannot be drawn from the present findings. Accordingly, the interpretation of these findings should consider both the magnitude of change (Δ) and the associated 95% confidence intervals.

### 4.3. Balance

Obesity has been associated with impaired balance control due to altered weight distribution and postural adaptations, which may influence functional stability [28]. Pilates has been reported to improve neuromuscular stability and postural control in various populations [9,19,21]. In the present study, both the Pilates circuit training group and the weight circuit training group demonstrated significant improvements in dynamic and static balance as assessed by the Y-Balance Test (YBT) and Balance Error Scoring System (BESS). The magnitude of change appeared greater in the PCG, consistent with previous reports indicating that Pilates may enhance balance through core activation and neuromuscular coordination [19,21,29].

Specifically, PCG demonstrated comparatively larger improvements in YBT performance for both right (d = 8.59) and left legs (d = 8.00), with between-group differences reaching statistical significance (*p* < 0.001; d = 0.52–1.13). Similarly, in static balance assessed by BESS, PCG showed larger within-group effect sizes on both firm (d = 3.33) and foam surfaces (d = 2.78) than WCG (d = 1.33 and 1.84, respectively), with between-group differences particularly evident on the firm surface (d = 1.54). However, given that BESS scoring relies on visual error counting by the assessor, and assessor blinding was not implemented, the observed improvements in static balance should be interpreted with caution due to the potential influence of detection bias.

Given the exploratory design and relatively small sample size, the observed effect sizes may be overestimated. Therefore, the findings should be considered preliminary and hypothesis-generating rather than confirmatory. In addition to the intervention effects, potential learning or familiarization effects associated with repeated exposure to balance assessments should be considered when interpreting the present findings. Both the Y-Balance Test and the Balance Error Scoring System (BESS) involve task-specific motor control and coordination, and performance may improve with repeated trials independent of physiological adaptations. Although familiarization trials were provided prior to baseline testing, it is possible that repeated testing across the intervention period contributed to improvements in performance. This factor may partially account for the observed within-group changes, particularly in dynamic balance measures. This interpretation is consistent with previous reports indicating that repeated exposure to balance and functional performance tests may lead to learning-related improvements independent of intervention effects [30,31]. Given the small sample size and exploratory design, the interpretation of statistical significance should be considered alongside the magnitude and variability of the observed effects, rather than as definitive evidence of intervention efficacy.

These findings may be associated with adaptations in neuromuscular coordination, trunk stability, and sensorimotor integration; however, these mechanisms were not directly assessed in the present study. Although impaired balance is a recognized risk factor for falls in middle-aged women with obesity, further large-scale randomized trials are required to determine whether these changes translate into meaningful functional improvements, including potential reductions in fall risk. Accordingly, the interpretation of these findings should consider both the magnitude of change (Δ) and the associated 95% confidence intervals.

### 4.4. Limitations of the Study

This study has several limitations that should be considered when interpreting the findings. These considerations are consistent with previous reports indicating that effect size estimates derived from small samples may be unstable and subject to inflation [32].

First, this study was designed as an exploratory randomized trial with a relatively small sample size (*n* = 9 per group). Although large effect sizes were observed, these estimates may be inflated due to the small sample size and should therefore be interpreted with caution and considered preliminary. The primary purpose of this study was to explore preliminary effects and to provide effect size estimates to inform sample size calculations for future large-scale randomized trials.

Second, the absence of assessor blinding may have introduced potential measurement bias. Although standardized assessment procedures were applied, this methodological constraint should be considered when interpreting the findings.

Third, the short intervention duration of eight weeks limits conclusions regarding the long-term sustainability of the observed improvements in body composition, pelvic alignment, and balance control. Longer intervention periods and follow-up assessments are needed to determine whether these effects are maintained over time.

Fourth, although participants were instructed to maintain their usual lifestyle habits, dietary intake, habitual physical activity, and other lifestyle factors were not strictly controlled, which may have influenced changes in body composition and related outcomes. Additionally, body composition was assessed using bioelectrical impedance analysis, which is sensitive to hydration status, and menstrual cycle phase was not controlled. Therefore, fluid fluctuations related to hormonal changes may have influenced body composition measurements, and these findings should be interpreted with caution. Future studies incorporating dietary monitoring, menstrual cycle tracking, and objective physical activity assessment would strengthen causal interpretation.

Fifth, potential learning effects and test familiarity associated with repeated balance assessments (Y-Balance Test and Balance Error Scoring System) cannot be excluded. While familiarization trials were provided, repeated exposure to these tests may have contributed to performance improvements independent of the intervention effects.

Sixth, pelvic alignment in this study was assessed using a three-dimensional surface topography system (Moti Physio), which estimates postural alignment based on external anatomical landmarks rather than direct skeletal imaging. Although such systems offer practical advantages in terms of non-invasiveness and feasibility, they do not represent a gold-standard method for assessing true skeletal alignment compared with radiographic or imaging-based techniques. Consequently, the validity of pelvic angle measurements may be influenced by factors such as soft tissue distribution, marker placement, and surface contour variability. Therefore, the present findings should be interpreted as reflecting functional postural alignment rather than precise structural pelvic alignment, and caution is warranted when extrapolating these results to anatomical or clinical interpretations. These limitations are consistent with previous reports indicating that surface-based postural assessment systems may differ from radiographic measures in estimating spinal and pelvic alignment [33].

Finally, exercise intensity was regulated using heart rate and rating of perceived exertion, which may not fully capture individual physiological load. In addition, maximum heart rate (HRmax) was estimated using an age-based predictive equation, which may introduce error, particularly in obese or sedentary individuals. Therefore, exercise intensity prescribed using heart rate reserve should be interpreted with caution.

Taken together, these limitations underscore the exploratory nature of the present study and highlight the need for larger, longer, and more methodologically rigorous trials to further clarify the effects and potential implications of Pilates- and resistance-based exercise interventions in middle-aged obese women.

## 5. Conclusions

This exploratory study compared the effects of Pilates circuit training and weight circuit training on body composition, pelvic alignment indices, and balance performance in middle-aged women with obesity. Both exercise modalities were associated with improvements in adiposity-related indicators and balance outcomes.

The findings suggest that Pilates circuit training may be associated with greater changes in selected pelvic alignment indices and balance measures, whereas weight circuit training may be associated with the preservation of lean body mass. However, these observations should be interpreted with caution due to the exploratory design and limited sample size.

Overall, the present results provide preliminary insights into potential differences between exercise modalities in this population. Rather than establishing definitive conclusions, these findings may serve as a basis for hypothesis generation and for guiding the design of future adequately powered randomized trials. Further research incorporating larger sample sizes, longer intervention periods, and comprehensive physiological assessments is required to better understand the robustness and potential relevance of these observations.

## Figures and Tables

**Table 1:** Characteristics of participants.

Group (N = 18)	Age (Years)	Height (cm)	Weight (kg)	% Body Fat
PCG (*n* = 9)	49.44 ± 2.06	160.33 ± 7.37	58.83 ± 3.29	32.88 ± 1.11
WCG (*n* = 9)	48.22 ± 3.29	159.66 ± 8.01	60.21 ± 2.48	33.88 ± 1.40

Values are Mean ± SD. PCG, Pilates circuit training group; WCG, weight circuit training group.

**Table 2:** Pilates and weight training program.

Mode	Muscle	Pilates Circuit (Reformer)	Machine-Based Weight Circuit
Push	Pectoralis major	Seated Chest Press	Seated Chest Press
Leg	Femoralis	Seated Leg Press	Plank with leg extension
Pull	Latissimus dorsi	Prone Long box Row	Seated Lat Pull Down
ABS	Rectus abdominis	Quadruped core stabilization	Machine-based abdominal crunch
Push	Deltoids	Prone Long box Shoulder	Seated Shoulder Press
Leg	Quadriceps femoris	Supine Longbox Leg Press	Seated Leg Extension
Pull	Biceps Brachii	Seated Bicep Curl	Seated Bicep Curl
ABS	Erector spinae	Back Extension	Back Extension
Push	Triceps	Seated Triceps Extension	Seated Triceps Extension
Leg	Biceps femoris	Prone Long box Leg Curl	Lying Leg Curl

**Table 3:** Body composition.

Variable	Group	Pre	Post	Δ (w)	95% CI	*p*	Cohen’s d
Body weight (kg)	PCG	58.83 ± 3.50	55.74 ± 3.34 ***	−3.09	−3.42 to −2.76	<0.001	PCG d(w): −0.90
	WCG	60.21 ± 2.63	58.21 ± 1.57 **	−2.00	−3.28 to −0.72	0.007	WCG d(w): −1.06
Between-group	-	-	-	−2.47	−5.08 to 0.14	0.062	d(b): −0.46
LBM (kg)	PCG	22.39 ± 1.54	21.80 ± 1.43	−0.59	−0.76 to −0.42	<0.001	PCG d(w): 0.06
	WCG	22.64 ± 1.51	23.08 ± 2.71	+0.43	−2.25 to 1.39	0.598	WCG d(w): 0.19
Between-group	-	-	-	−1.28	−3.44 to 0.89	0.229	d(b): −0.21
Fat mass (kg)	PCG	19.39 ± 1.40	17.33 ± 1.38 ***	−2.06	−2.29 to −1.82	<0.001	PCG d(w): −1.47
	WCG	20.27 ± 0.94	18.86 ± 1.01 **†	−1.41	−2.20 to −0.63	0.003	WCG d(w): −1.47
Between-group	-	-	-	−1.52	−2.73 to −0.31	0.017	d(b): −0.48
Body fat %	PCG	32.89 ± 1.19	31.04 ± 1.55 ***	−1.84	−2.29 to −1.40	<0.001	PCG d(w): −1.26
	WCG	33.89 ± 1.49	32.36 ± 1.67 ***	−1.53	−2.19 to −0.88	0.001	WCG d(w): −0.95
Between-group	-	-	-	−1.31	−2.92 to 0.30	0.104	d(b): −0.94
WHR	PCG	0.88 ± 0.02	0.83 ± 0.01 ***	−0.05	−0.06 to −0.04	<0.001	PCG d(w): −2.70
	WCG	0.90 ± 0.03	0.86 ± 0.03 ***††	−0.04	−0.05 to −0.02	0.001	WCG d(w): −1.28
Between-group	-	-	-	−0.04	−0.06 to −0.02	0.002	d(b): −0.93

Values are presented as mean ± standard deviation (SD). Δ indicates the mean difference (post − pre). 95% CI denotes the 95% confidence interval of the mean difference. LBM, lean body mass; WHR, waist-to-hip ratio; PCG, Pilates circuit training group; WCG, weight circuit training group; d(w), within-group effect size; d(b), between-group effect size. Significantly different between pre- and post-intervention: ** *p* < 0.01, *** *p* < 0.001; significantly different between PCG and WCG: † *p* < 0.05, †† *p* < 0.01. Cohen’s d values are reported with their original sign to reflect the direction of change, and direction is additionally described in the text. Between-group values represent differences at post-intervention.

**Table 4:** Pelvic alignment indices.

Variable	Group	Pre	Post	Δ	95% CI	*p*	Cohen’s d
Pelvic tilt (**°**)	PCG	3.69 ± 0.45	2.16 ± 0.72 ***	−1.53	−1.98 to 1.08	<0.001	PCG d(w): −2.57
	WCG	3.31 ± 0.65	3.09 ± 0.42 ††	−0.22	−0.83 to 0.38	0.422	WCG d(w): 0.33
Between-group	-	-	-	−0.93	−1.52 to −0.35	0.004	d(b): −3.06
Pelvic rotation (**°**)	PCG	4.68 ± 0.37	3.21 ± 0.75 ***	−1.47	−2.06 to −0.87	<0.001	PCG d(w): −2.35
	WCG	4.44 ± 0.93	3.98 ± 0.35 †	−0.47	−1.36 to 0.43	0.263	WCG d(w): −0.59
Between-group	-	-	-	−0.77	−1.35 to −0.18	0.013	d(b): −1.36
Pelvic inclination (°)	PCG	8.60 ± 0.59	6.36 ± 1.34 **	−2.24	−3.36 to −1.13	0.002	PCG d(w): −2.11
	WCG	8.00 ± 0.95	7.64 ± 0.79 †	−0.36	−1.24 to 0.53	0.383	WCG d(w): −0.41
Between-group	-	-	-	−1.29	−2.39 to −0.19	0.024	d(b): −2.01

Values are Mean ± SD. Δ indicates the mean difference (post − pre), and 95% CI denotes the 95% confidence interval of the mean difference. PCG, Pilates circuit training group; WCG, weight circuit training group; d(w), within-group; d(b), between-group. Significantly different between pre vs. post at ** *p* < 0.01, *** *p* < 0.001; significantly different between PCG vs. WCG at † *p* < 0.05, †† *p* < 0.01. Cohen’s d values are reported with their original sign to reflect the direction of change. Direction is additionally described in the text. Between-group values represent differences at post-intervention.

**Table 5:** Balance test.

Variable	Group	Pre	Post	Δ	95% CI	*p*	Cohen’s d
Y-balance Right	PCG	73.34 ± 0.92	84.87 ± 1.66 ***	11.53	10.51 to 12.56	<0.001	PCG d(w): 8.94
	WCG	73.45 ± 0.89	82.65 ± 1.38 ***††	9.19	8.48 to 9.91	<0.001	WCG d(w): 8.11
Between-group	-	-	-	2.23	0.70 to 3.75	0.007	d(b): 0.52
Y-balance Left	PCG	73.33 ± 1.16	84.46 ± 1.59 ***	11.13	10.05 to 12.21	<0.001	PCG d(w): 8.10
	WCG	73.48 ± 1.27	81.50 ± 1.43 ***†††	8.02	9.16 to 6.88	<0.001	WCG d(w): 8.11
Between-group	-	-	-	2.95	1.44 to 4.47	0.001	d(b): 1.13
BESS firm	PCG	3.67 ± 0.87	1.00 ± 0.71 ***	−2.67	−3.44 to −1.90	<0.001	PCG d(w): −3.33
	WCG	3.00 ± 1.22	1.56 ± 1.13 ***	−1.44	−1.85 to −1.04	<0.001	WCG d(w): −1.33
Between-group	-	-	-	−0.56	−1.50 to 0.39	0.229	d(b): −1.54
BESS foam	PCG	11.78 ± 1.72	7.33 ± 1.50 ***	−4.44	−5.22 to −3.67	<0.001	PCG d(w): −2.78
	WCG	11.89 ± 2.15	8.56 ± 0.88 ***	−3.33	−4.77 to −1.90	0.001	WCG d(w): −1.84
Between-group	-	-	-	−1.22	−2.45 to 0.01	0.051	d(b): −0.62

Values are Mean ± SD. Δ indicates the mean difference (post − pre), and 95% CI denotes the 95% confidence interval of the mean difference. BESS, Balance error scoring system. PCG, Pilates circuit training group; WCG, weight circuit training group; d(w), within-group; d(b), between-group. Significantly different between pre vs. post at *** *p* < 0.001; significantly different between PCG vs. WCG at †† *p* < 0.01, ††† *p* < 0.001. Cohen’s d values are reported with their original sign to reflect the direction of change. Direction is additionally described in the text. Between-group values represent differences at post-intervention.

## Data Availability

The data presented in this study are available from the corresponding author upon reasonable request. The data are not publicly available due to privacy and ethical restrictions.

## Supplementary Materials

The following supporting information can be downloaded at https://www.mdpi.com/article/10.3390/jfmk11020141/s1. Table S1. Between-group effect sizes, post-hoc power, and magnitude interpretation. Table S2. Partial η^2^ values for all outcome variables from two-way mixed ANOVA (Df1 = 1, Df2 = 16).
