# Supplementary material for: An Exploratory Comparison of Pilates and Weight Circuit Training on Body Composition, Pelvic Alignment, and Balance in Obese Middle-Aged Women
Source: J Funct Morphol Kinesiol. 2026 Mar 27;11(2):141. doi: 10.3390/jfmk11020141 (PMC13108094; doi:10.3390/jfmk11020141)
Supplement: Supplementary file 1 [file jfmk-11-00141-s001.zip › jfmk-4176922-supplementary.pdf]

Supplementary Table S1. Between-group effect sizes, post-hoc power, and magnitude interpretation.

Variable	Cohen's d(b)	Post-hoc Power	Effect Size Interpretation
**Pelvic tilt (°)**	-3.06	100.00%	Large
**Pelvic inclination (°)**	-2.01	97.90%	Large
**BESS firm (points)**	-1.54	86.60%	Large
**Pelvic rotation (°)**	-1.36	77.30%	Large
**Y-balance Left (%)**	1.13	61.50%	Large
**% Body fat**	-0.94	46.60%	Large
**WHR**	-0.93	45.80%	Large
**BESS foam (points)**	-0.62	23.60%	Medium
**Y-balance Right (%)**	0.52	18.00%	Medium
**Fat mass (kg)**	-0.48	16.00%	Small
**Weight (kg)**	-0.46	15.10%	Small
**LBM (kg)**	-0.21	7.00%	Small

Cohen's d values are reported as magnitudes (absolute). Direction of change is indicated in the text.

Supplementary Table S2. Partial η² values for all outcome variables from two-way mixed ANOVA (Df1 = 1, Df2 = 16).

Variable	F (time)	Partial η² (time)	F (group)	Partial η² (group)	F (time x group)	partial η² (time x group)
**Weight (kg)**	8.025	0.334	4.366	0.214	0.333	0.02
**LBM (kg)**	0.184	0.011	1.55	0.088	0.071	0.004
**Fat mass (kg)**	18.741	0.502	10.575	0.398	0.664	0.04
**% Body fat**	13.58	0.459	6.019	0.273	0.101	0.006
**WHR**	31.454	0.663	14.902	0.482	0.923	0.055
**Pelvic tilt (°)**	8.722	0.353	2.251	0.123	13.588	0.459
**Pelvic rotation (°)**	8.317	0.342	1.586	0.09	5.926	0.27
**Pelvic inclination (°)**	6.796	0.298	1.203	0.07	10.019	0.385
**Y-balance Right (%)**	614.828	0.975	7.093	0.307	8.829	0.356
**Y-balance Left (%)**	436.934	0.965	11.276	0.413	13.588	0.459
**BESS firm (points)**	37.766	0.702	0.028	0.002	3.572	0.183
**BESS foam (points)**	51.444	0.763	1.571	0.089	1.085	0.064

Note: For variables with $p < .001$, actual F-values from the SPSS ANOVA output were used to compute partial η^2 using the formula: partial $\eta^2 = (F \times df_1) / ((F \times df_1) + df_2)$ where F is the actual ANOVA statistic, $df_1 = 1$, and $df_2 = 16$. Large F-values may yield partial η^2 values close to 1.0, indicating near-total variance explanation.
